# Supplementary material for: Photon Counting CT Angiography of the Head and Neck: Image Quality Assessment of Polyenergetic and Virtual Monoenergetic Reconstructions
Source: Diagnostics (Basel). 2022 May 24;12(6):1306. doi: 10.3390/diagnostics12061306 (PMC9222087; doi:10.3390/diagnostics12061306)
Supplement: Supplementary file 1 [file diagnostics-12-01306-s001.zip › diagnostics-1712350-supplementary.pdf]

Photon Counting CT Angiography of the Head and Neck: Image Quality Assessment of Polyenergetic and Virtual Monoenergetic Reconstructions

Arwed Elias Michael [1,*], Jan Boriesosdick [1], Denise Schoenbeck [1], Ingo Lopez-Schmidt [1], Jan Robert Kroeger [1], Christoph Moenninghoff [1], Sebastian Horstmeier [1], Lenhard Pennig [2], Jan Borggrefe [1] and Julius Henning Niehoff [1]

[1] Department of Radiology, Neuroradiology and Nuclear Medicine, Johannes Wesling University Hospital, Ruhr University Bochum, Bochum, Germany;Jan.Boriesosdick@muehlenkreiskliniken.de (J.Bs.); Denise.Schoenbeck@muehlenkreiskliniken.de (D.S.); Ingo.Lopez-Schmidt@muehlenkreiskliniken.de (I.L.-S.); Christoph.Moenninghoff@muehlenkreiskliniken.de (C.M.); Sebastian.Horstmeier@muehlenkreiskliniken.de (S.H.); JanRobert.Kroeger@muehlenkreiskliniken.de (J.R.K.); Jan.Borggrefe@muehlenkreiskliniken.de (J.B.); Julius.Niehoff@muehlenkreiskliniken.de (J.H.N.)

[2] Institute of Diagnostic and Interventional Radiology, Faculty of Medicine and University Hospital Cologne, University of Cologne, Cologne, Germany; Lenhard.Pennig @uk-koeln.de

* Correspondence: arwed.michael@muehlenkreiskliniken.de; Tel.: +0049-571-790-4601

Supplementary Material

In particular, it is interesting to compare the results of our study with those of Euler et al.[1] who investigate the image quality of polyenergetic reconstruction (PER) and some monoenergetic reconstructions (MER) from the low keV range in CT angiographies of the aorta in the same photon counting CT (NAEOTOM Alpha; Siemens Healthcare GmbH, Forchheim, Germany).

In their study, Euler et al. measured the signal, defined as attenuation in hounsfield units (HU), at different locations in the aorta as well as the common iliac artery. The signal in the aorta in the PER and MER strongly resembles the signal in the extracranial vessels in our study, both in absolute values and across the different reconstructions, as shown in table 1 below:

reconstruction	*signal aorta (Euler et al.)*	*signal extracranial arteries*
PER	302 ± 65 HU	377,15 ± 78,26 HU
MER 40 keV	873 ± 202 HU	937,55 ± 204,71 HU
MER 45 keV	711 ± 163 HU	762,59 ± 165,06 HU
MER 50 keV	584 ± 133 HU	625,14 ± 133,87 HU
MER 55 keV	486 ± 109 HU	518,35 ± 109,63 HU

Table S1: Signal in the aorta (Euler et al.) and signal in the extracranial vessels in the present study compared.

The noise is defined by Euler et al. as follows: "[...] CT attenuation and its standard deviation were measured 4 times in the psoas muscle at the level of the lower pole of the right kidney. [...] The average of the standard deviation of the CT attenuation of the psoas muscle was used as image noise." Consistent with the studies on CT angiographies of the head and neck [e. g. 2], noise is defined differently in our study: "Signal was defined as the average density of voxels of the ROIs in Hounsfield units (HU), and noise was defined as the standard deviation (SD) of all voxels of the ROI." Signal and noise correlate with each other, so that with the significantly higher signal in the contrasted vessels, a higher noise is also measured. A noise measured in the musculature is small in relation. Moreover, it increases less with stronger contrast than the noise in the contrasted vessel itself.

Also in our study, a ROI was measured in a muscle, namely the lateral pterygoid muscle, so we can express the noise similarly to Euler et al. as the standard deviation of a ROI in the muscle. table 2 below shows the comparison of the noise, figure 1 shows the comparison of the noise of the air and the muscle in our study.

reconstruction	*noise (sd psoas muscle) Euler et al.*	*noise (pterygoid muscle) current study*	*noise (air) current study*
PER	22 ± 4 HU	10,59 ± 1,76 HU	5,59 HU
MER 40 keV	36 ± 6 HU	21,78 ± 3,52 HU	15,03 HU
MER 45 keV	32 ± 5 HU	19,46 ± 3,06 HU	13,41 HU
MER 50 keV	29 ± 5 HU	17,41 ± 2,67 HU	12,14 HU
MER 55 keV	28 ± 4 HU	15,70 ± 2,56 HU	10,92 HU

Table S2: Noise defined as the standard deviation of a ROI in the muscle in the CT scan. Comparison of Euler et al. (psoas muscle in CT angiography of the aorta) and current study (air or lateral pterygoid muscle in CT angiography of the head and neck).

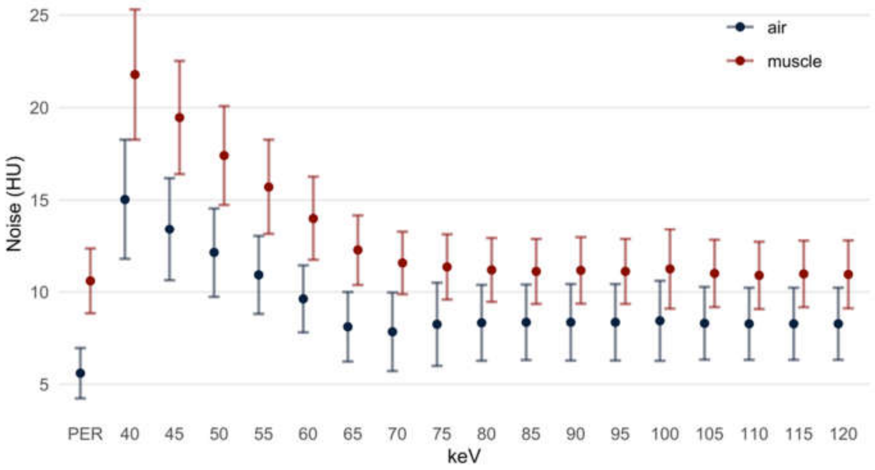

Figure S1: Comparison of noise in ROI of air and ROI of muscle in our study.
HU: Hounsfield units. PER: polyenergetic reconstruction.
keV: kilo electron volt of the monoenergetic reconstruction.

The significantly lower absolute values for noise measured in muscle in our study can be explained by the different dose (CTDIvol in Euler et al. 2.63 ± 1.3 mGy; CTDIvol in our study 8.31 ± 1.19 mGy) and the different body region in each study (aorta in thorax and abdomen vs. extracranial vessels of the neck). Moreover, both studies applied different reconstruction parameters, e.g. slice thickness, kernel and level of quantum iterative reconstruction which all affect noise. The ratio between the different reconstructions appears very similar. Note that the ratio of noise in muscle is different from that in air: in air, the noise in the 40 keV is about 3 times that in PER; in muscle, it is about a factor of 2.

These different ratios also explain the discrepancies in the contrast-to-noise ratio (CNR) in the study by Euler et al. and our study. Euler et al. defined the CNR as follows:

$$CNR = \frac{signal_{artery} - signal_{muscle}}{standard\ deviation_{muscle}}$$

In our study, in agreement with previous studies of image quality in CT angiography of the head and neck, the definition was different:

$$CNR = \frac{signal_{artery} - signal_{muscle}}{standard\ deviation_{air}}$$

Thus, in Euler et al. the noise from the muscle is included in the CNR, in our study the noise from the immediately surrounding air. Since the ratios of the different reconstructions differ significantly for the noise in muscle and air (factor 2 vs. factor 3, see above), this results in a different outcome for the CNR.

In the following table 3, the CNR for our data has additionally been calculated according to the definition of Euler et al. figure 2 shows the comparison of the CNR once calculated with the noise from the air, the other time with the noise from the muscles.

reconstruction	*cnr (noise muscle) Euler et al.*	*cnr (noise muscle) current study*	*cnr (noise air) current study*
PER	12 ± 4	30,41 ± 10,00	58,72 ± 19,90
MER 40 keV	22 ± 7	39,54 ± 11,86	59,01 ± 22,68
MER 45 keV	20 ± 6	35,51 ± 10,47	52,95 ± 20,05
MER 50 keV	18 ± 5	32,13 ± 9,65	46,92 ± 16,37
MER 55 keV	16 ± 5	29,23± 9,19	42,55 ± 15,09

Table S3: CNR calculated with the noise of the muscle and the air, respectively. Comparison of Euler et al. (psoas muscle in CT angiography of the aorta) and current study (air or lateral pterygoid muscle in CT angiography of head and neck).

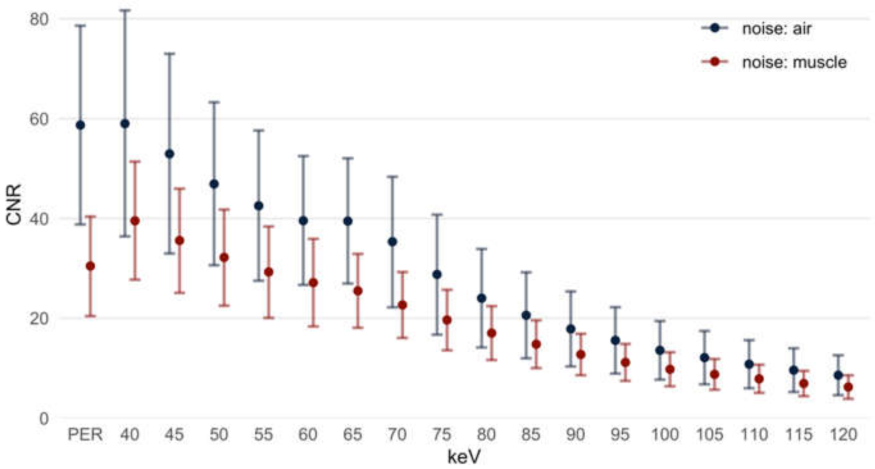

Figure S2: Comparison of the CNR with different calculation: one time with the noise from the air, the other time with the noise from the musculature. HU: Hounsfield units. PER: polyenergetic reconstruction. keV: kilo electron volt of the monoenergetic reconstruction.

Eventually, both calculations are possible and reasonable. The calculation of the CNR using the noise from the air ultimately leads to the fact that the overall impression from the qualitative analysis is also well represented in the quantitative data.

Literature

1. Euler, A.; Higashigaito, K.; Mergen, V.; Sartoretti, T.; Zanini, B.; Schmidt, B.; Flohr, T.G.; Ulzheimer, S.; Eberhard, M.; Alkadhi, H. High-Pitch Photon-Counting Detector Computed Tomography Angiography of the Aorta: Intraindividual Comparison to Energy-Integrating Detector Computed Tomography at Equal Radiation Dose. *Invest Radiol* **2022**, *57*, 115-121, doi:10.1097/rli.0000000000000816.
2. Neuhaus, V.; Abdullayev, N.; Große Hokamp, N.; Pahn, G.; Kabbasch, C.; Mpotsaris, A.; Maintz, D.; Borggrefe, J. Improvement of Image Quality in Unenhanced Dual-Layer CT of the Head Using Virtual Monoenergetic Images Compared With Polyenergetic Single-Energy CT. *Invest Radiol* **2017**, *52*, 470-476, doi:10.1097/rli.0000000000000367.
